# Somatic genomic profiling reveals clinically relevant heterogeneity in *RAS*-mutant sporadic medullary thyroid carcinoma

**DOI:** 10.1016/j.jcte.2026.100442

**Published:** 2026-04-28

**Authors:** Edoardo Ruggeri, Simona Censi, Loris Bertazza, Andrea Benetti, Fausto Cortese, Ilaria Piva, Cristina Clausi, Vincenzo Marotta, Francesca Galuppini, Alessandra Giannella, Gianmaria Pennelli, Maurizio Iacobone, Mario Vitale, Giulio Ceolotto, Susi Barollo, Caterina Mian

**Affiliations:** a Department of Medicine (DIMED), University of Padua, Padua, Italy; b Endocrinology Unit, University Hospital of Padua, Padua, Italy; c Thrombotic and Hemorrhagic Diseases Unit, University Hospital of Padua, Padua, Italy; d Veneto Institute of Oncology IOV - IRCCS, Padua, Italy; e Department of Clinical Medicine and Surgery, University of Salerno, Italy; f Surgical Pathology & Cytopathology Unit, University Hospital of Padua, Padua, Italy; g Internal Emergency Medicine Unit, University Hospital of Padua, Padua, Italy; h Department of Surgical, Oncological and Gastroenterological Sciences (DiSCOG), University of Padua, Padua, Italy; i Endocrine Surgery Unit, University Hospital of Padua, Padua, Italy

**Keywords:** NGS somatic panel, RAS-mutant tumors, Precision medicine strategies, T status, Biochemical cure

## Abstract

**Objective:**

Sporadic medullary thyroid carcinoma (sMTC) is predominantly driven by somatic *RET* or *RAS* mutations, although the molecular basis of disease heterogeneity remains incompletely understood. Our study aims to characterize molecular heterogeneity in sMTC in order to identify pathways that may improve patient’s stratification and support more personalized clinical management strategies.

**Methods:**

Deep targeted next-generation sequencing of 31 neuroendocrine- and cancer-related genes was performed on tumor samples from 94 patients with sMTC to characterize the somatic mutational landscape beyond canonical drivers.

**Results:**

*RET* and *RAS* mutations were detected in 53% and 29% of cases, respectively, while 18% of tumors lacked both alterations. Variant allele frequency (VAF) analysis demonstrated a significant positive correlation between driver mutation burden and tumor size in both *RET*- and *RAS*-mutant tumors, supporting the clonal contribution of these alterations. Additional oncogenic variants were identified in genes involved in DNA damage response and epigenetic regulation, including *ATM* and *KMT2A*. Notably, within the *RAS*-mutant subgroup, the presence of co-occurring oncogenic alterations was associated with more advanced T status (T3-T4, *p* = 0.0181) at diagnosis and lower biochemical cure rates (*p* = 0.02) at the follow-up compared with tumors harboring isolated *RAS* mutations, supporting the clinical relevance of extended genomic profiling in *RAS*-mutant sMTC.

**Conclusions:**

Overall, these findings highlight additional oncogenic alterations potentially involved in tumor progression and suggest that extended targeted profiling may provide clinically relevant information on molecular heterogeneity in sMTC, particularly within *RAS*-mutant tumors.

## Introduction

Medullary thyroid carcinoma (MTC) is a rare neuroendocrine malignancy deriving from thyroid C cells [1]. MTC accounts for approximately 2–3% of all thyroid neoplasms yet is responsible for more than 13% of thyroid cancer-related deaths [2]. Ten-year survival rates decline markedly with advancing stage, ranging from 100% in stage I to 21% in stage IV [1]. In its absence, either biochemical or structural disease may persist, with 20-year disease-specific mortality rates of 15% and 75%, respectively [3]. The clinical course of MTC is highly variable: while some patients may live for decades with low-volume nodal disease, others experience rapid progression and succumb quickly to aggressive metastatic disease [4], [5]. The molecular mechanisms underlying this striking heterogeneity remain poorly understood. The mutational status of the rearranged during transfection (*RET*) proto-oncogene plays a fundamental role in driving MTC aggressiveness and patient outcomes [6], [7].

Approximately 75% of MTC cases are sporadic, while the remaining 25% are inherited as part of the multiple endocrine neoplasia types 2A and 2B (MEN2A and MEN2B) caused by *RET* germline mutations [8]. Nearly all familial cases, along with 40–60% of sporadic MTC (sMTC) cases, originate from *RET* mutations [6], [9]. Moreover, mutually activating point mutations in the Rat Sarcoma (*RAS*) virus GTPase gene family (*H*-, *K*-, and *N-RAS*) underlie approximately 20–40% of *RET*-wildtype sMTC cases^6^. Rather surprisingly, the oncogenic mechanisms driving the remaining subset of sMTCs, as well as certain rare familial cases, remain poorly understood. Emerging molecular targets in addition to *RET* and *RAS* mutations may open new therapeutic possibilities [10].

This study employed a targeted next-generation sequencing (NGS) approach based on a neuroendocrine-focused 31-gene panel to characterize the somatic mutational landscape of sMTC. By extending the analysis beyond established driver mutations, the study aimed to explore molecular heterogeneity and provide additional insight into pathways potentially relevant for refining patient stratification and informing more tailored clinical management strategies.

## Materials & methods

### Sample collection

The study followed the Declaration of Helsinki and retrospectively analyzed 111 sMTC specimens at the University of Padua from 2004 to 2022. Seventeen cases were excluded due to insufficient quality, leaving 94 informative samples. Sporadic status was confirmed by the absence of germline RET mutations, systematically screened by Sanger sequencing of exons 5, 8, 10, 11, 13–16. Somatic driver mutations were validated by Sanger sequencing on tumor tissue [6].

### Patients

Pathology reports were reviewed using the American Joint Committee on Cancer (AJCC) 8th edition TNM staging. All 94 patients were followed at the Endocrinology Unit, University of Padua. Medical records were retrospectively reviewed to collect data on patients’ demographics (including age, sex, and date of surgery), histopathological features, and disease status (Table 1). Outcomes were classified as: excellent response (undetectable calcitonin (Ct) and normal Carcinoembryonic antigen (CEA) levels, no structural disease), biochemical incomplete response (detectable Ct or high CEA levels, no structural disease), structural disease, or disease-related death (any death in which MTC had played a causally relevant role). To classify MTC grading, we applied the International Medullary Thyroid Carcinoma Grading System (IMTCGS), which categorizes tumors as high grade when at least one of the following features is present: tumor necrosis, a mitotic count ≥5 per 2 mm2, and/or a Ki-67 proliferation index ≥5% [11].

**Table 1:** Clinicopathological features of sMTC patients.

Characteristics	All cases (N. = 94)
Sex, (F%:M%)	59:35
Age at diagnosis: median; IQR	60; 51.6 – 66.7
Primary tumor size, (cm): median; range; IQR.	1.6; 0.3–7; 1.1 – 2.5
AJCC stage (%)	
I	39 (41.5)
II	20 (21.3)
III	10 (10.7)
IV	25 (26.5)
T (%)	
1	50 (53.2)
2	21 (22.3)
3	21 (22.3)
4	2 (2.2)
N (%)	
0	60 (64)
1	34 (36)
M (%)	
0	85 (90.4)
1	9 (9.6)
Follow-up, months: Median; IQR	83.5; 50-123
IMTCGS grading (%)	
Low-grade	74 (79)
High-grade	20 (21)
Outcome (%)	
Disease-free	47 (50)
Incomplete response	39 (41.5)
Disease-related death	8 (8.5)
Mutational status RET	
RET-mutated (%) / RET-wilde-type (%)	50 (53) / 44 (47)
RET-M918T (%)	24/50 (48 )
Mutational status RAS (%)	27 (29)
Non-RET/non-RAS −mutated	17 (18)

### Sample preparation and histopathological assessment

Primary sMTC tumor samples were snap-frozen after surgery, with H&E–stained sections reviewed by pathologists (G.P. and F.G.) at the University Hospital of Padua to confirm diagnosis and estimate tumor cellularity (>70% mean). Only primary tumors were analysed. About 25 mg of tumor tissue per case was collected for DNA extraction using the Tissue RNA/DNA Purification Kit Norgen Biotek (Thorold, Canada), following the manufacturer’s instructions, and quality and yield were assessed fluorometrically. Sample purity was further evaluated by qRT-PCR for Ct (C-cell marker) and thyroglobulin (follicular marker). Procedures are detailed in Supplementary Methods (Table S1).

### NGS gene panel set-up

A targeted 31-genes NGS panel was used to detect low-frequency somatic single nucleotide variants (SNVs) and indels in MTC samples, achieving 1,000 × depth and >99.9% coverage. Probes were designed with IDT xGen™ (Supplementary Table S2).

### NGS library preparation

Library preparation was performed using the xGen™ DNA Library Prep EZ Kit (IDT, Tema Ricerca, Milan, Italy) starting from 100 ng of tumor DNA, including enzymatic fragmentation, end repair/A-tailing, adapter ligation, and PCR amplification with unique dual indexes. Libraries were purified using SPRI beads, quantified with the Qubit 4.0 Fluorometer (Thermo Fisher Scientific, Waltham, MA, USA), and assessed for fragment size distribution and quality using the LabChip GX system (Caliper Life Sciences, Hopkinton, MA, USA).

### Target enrichment

Target enrichment was performed using the xGen™ Hybridization Capture of DNA Libraries Kit (IDT) following the manufacturer’s protocol. Libraries were hybridized to biotinylated, target-specific probes and captured with streptavidin-coated magnetic beads, followed by stringent washes. The enriched DNA was then amplified with the xGen™ Library Amplification Primer Mix (IDT), purified, quantified, and quality-checked prior to sequencing.

### Library sequencing

Pooled libraries were denatured and diluted to 1.5 pM according to the manufacturer’s instructions, with 1% PhiX Control DNA (Illumina, San Diego, CA, USA) added as an internal sequencing control. Libraries were denatured with 0.2 N NaOH, neutralized with Tris-HCl, diluted in HT1 buffer, and sequenced on the NextSeq 550 System (Illumina) using a paired-end Mid Output Kit (300 cycles). The use of unique molecular identifiers (UMIs) enabled error correction and reliable detection of somatic SNVs down to a 2% variant allele frequency [12].

### Bioinformatic analysis

Raw sequencing data (FASTQ format) underwent quality control assessment, including evaluation of read quality scores, GC content, sequence duplication levels, and adapter contamination. Low-quality bases and adapter sequences were trimmed and reads failing quality thresholds were excluded from downstream analysis. Sequencing performance was evaluated using standard metrics, such as % ≥Q30 (Q30 = 91.1 ± 2), cluster density (221 ± 4 k/mm^2^), and percentage of passing filter reads (%PF = 90.7 ± 0.25). Only samples passing quality control criteria were retained for downstream analysis. High-quality reads were analyzed using CLC Genomics Workbench (v25.0.1, Qiagen), with reads aligned to the GRCh38/hg38 reference genome. Variant calling was performed using dedicated germline and somatic workflows, applying variant allele frequency (VAF) thresholds and low-frequency variant detection (<2%), followed by filtering to remove artifacts. Filtered variants and BAM files were visualized using IGV (Integrative Genomics Viewer).

### Validation and interpretation of genetic variants

Genetic variants affecting coding regions or splicing sites were filtered and classified according to ACMG/AMP guidelines, following the somatic variant interpretation framework proposed by Horak et al. [13]. Classification was supported by bioinformatics tools and databases including ClinVar, Franklin (Genoox), VarSome, and COSMIC (v102, released 1-May-25). Variants were categorized as Oncogenic, Likely Oncogenic, VUS, Likely Benign, or Benign, and only Oncogenic, Likely Oncogenic, and VUS variants were included in the study.

### Statistical analyses

Statistical analyses were conducted using GraphPad Prism 10 (version 10.4.2, GraphPad Software, Boston, Massachusetts, USA) and R software (version 3.0.3, R Foundation for Statistical Computing, Vienna, Austria). Data normality was assessed with the Kolmogorov–Smirnov test and, due to non-normal distributions, results are reported as medians and IQRs. Categorical variables were compared using the Chi-square test, while nonparametric tests (Mann–Whitney U and Kruskal–Wallis) were applied for quantitative variables that were not normally distributed. Survival analyses were performed using Kaplan–Meier curves with log-rank tests, followed by Bonferroni-corrected post hoc analyses when appropriate, and multivariable Cox regression. Linear regression was used to evaluate the correlation between VAF and tumor size, with *p*-value < 0.05 considered statistically significant.

## Results

### Genetic distribution of known driver mutations in the sMTC cohort

**Fig. 1:**
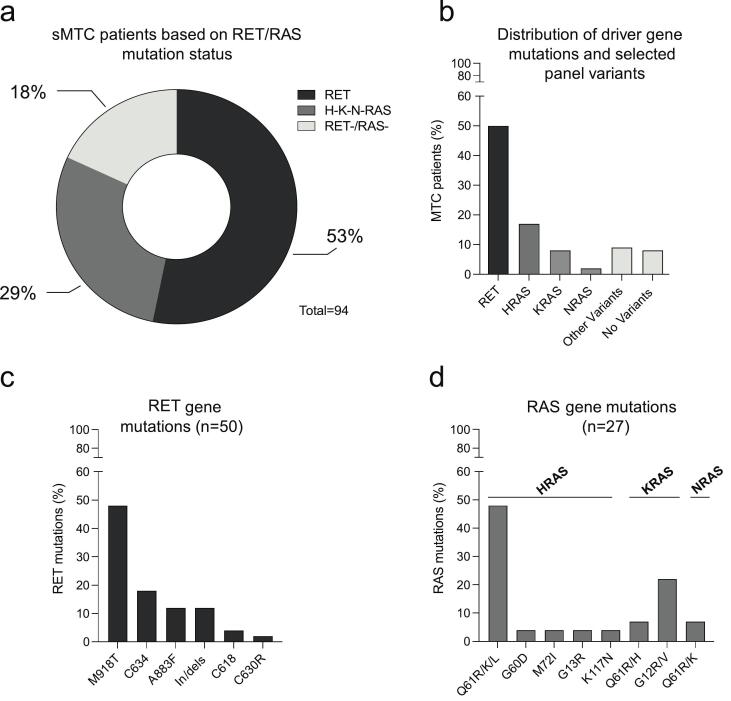
Distribution of driver mutations and associated somatic variants in the sMTC cohort. (a) Pie chart showing the distribution of 94 sporadic MTC cases based on driver mutation status: *RET*-positive, *RAS*-positive, and *RET*/*RAS*-negative. (b) Histogram illustrating the mutational landscape across the cohort, including cases harboring *RET* or *RAS* driver mutations, additional non-driver somatic variants in panel genes, or no detectable alterations. (c) Overview of *RET* gene alterations identified in *RET*-positive patients. (d) Distribution of gene alterations detected in RAS-positive patients among the three isoforms *HRAS*, *KRAS*, and *NRAS*.

Based on molecular profiling, MTC patients were stratified into RET-positive, RAS-positive, and RET/RAS-negative groups (Fig. 1a). RET driver mutations were detected in 53% (50/94) of cases, RAS mutations in 29% (27/94), while the remaining 18% included patients with other gene alterations (10%) or no detectable somatic variants (8%) (Fig. 1b).

The distribution of somatic *RET* mutations is shown in Fig. 1c. The most frequent alteration was M918T in exon 16 (48%, 24/50), followed by C634 mutations in exon 11 (18%, 9/50). A883F mutation and insertions/deletions were each detected in 12% (6/50) of cases, while less frequent mutations involved amino acid C618 (4%, 2/50) and C630R (2%, 1/50).

Among *RAS* genes, *HRAS* mutations were the most frequent (18%, 17/94), followed by *KRAS* (9%, 8/94) and *NRAS* (2%, 2/94). The most common *HRAS* variants were Q61R, Q61K, and Q61L in exon 3, accounting for 48% of all *RAS* mutations. Other *HRAS* alterations included G60D and M72I (exon 3), G13R (exon 2), and K117N (exon 4), each detected in a single case (altogether 4%). *KRAS* mutations mainly affected exon 2 (G12R, G12V), with some exon 3 variants (Q61R, Q61H). *NRAS* mutations (Q61R, Q61K) were each found in one case (Fig. 1d).

### Somatic variant landscape identified through targeted NGS

**Fig. 2:**
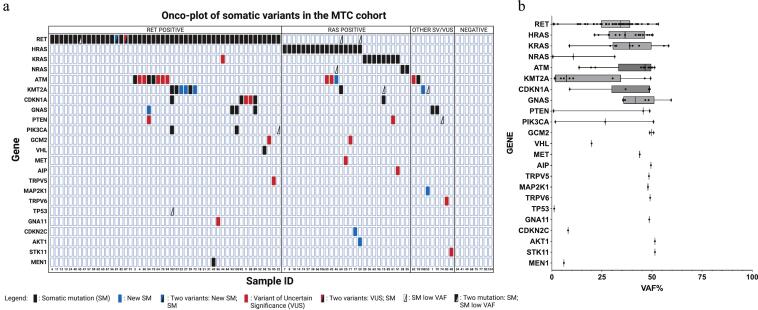
Mutational landscape of the MTC cohort based on targeted NGS analysis. (a) Oncoplot depicting the classification of 94 sporadic MTC cases. Each horizontal lane represents a single gene and each column an individual patient. Cases are divided into three molecular subgroups: *RET*-positive, *RAS*-positive, and *RET*/*RAS*-negative (the latter further subdivided into cases harboring additional somatic mutations or with no detectable alterations). Somatic variants are annotated according to predicted pathogenicity: black squares indicate known pathogenic mutations; blue squares, putative pathogenic variants not previously reported; and red squares, variants of uncertain significance (VUS). Black triangles mark pathogenic variants with a variant allele frequency (VAF) below 5%. (b) Box-and-whisker plot depicting the distribution of VAFs for all somatic variants identified across the targeted gene panel in the sMTC cohort.

The somatic variant landscape of the MTC cohort is shown in the oncoplot (Fig. 2a). Among the 94 sMTC patients, 140 variants were identified, including 124 SNVs, 11 indels, and 5 frameshift mutations. Among these, 109 were oncogenic, including 10 novel alterations (8.6%), while 21 variants (13.6%) were classified as VUS. Somatic mutations in *RET* and *RAS* genes represented 60.7% of all variants (85/140), confirming their key role in MTC. Recurrent alterations (oncogenic and VUS) involved *ATM* (9%, 13/140), *KMT2A* (7%, 10/140), *CDKN1A* and *GNAS* (4% each, 6/140), *PTEN* and *PIK3CA* (2% each, 3/140), and *GCM2* (1.4%, 2/140). Single variants (0.7% each, 1/140) were detected in *VHL*, *MEN1*, *MET*, *AIP*, *TRPV5*, *MAP2K1*, *TRPV6*, *TP53*, *GNA11*, *CDK2C*, *AKT1*, and *STK11*. No oncogenic variants were found in *CDKN1B*, *CDKN2B*, *CASR*, *CDC73*, *KMT2C*, *EIF1AX*, *CTNNB1*, or *CDKN2D*; all detected variants in these genes were classified as likely benign or known polymorphisms. Fig. 2b shows VAF distribution, highlighting marked inter-gene heterogeneity. Several low-VAF oncogenic variants co-occurred with high-VAF drivers. Two patients with high-VAF *HRAS* mutations also carried low-frequency *RET* M918T variants (VAF < 2%). Additional co-occurring events included *RET* D631E in a tumor with *RET* P496_D499del, *RET* S891A in a *RET* C634Y tumor, and *RET* D567N (exon 9) or T1038A (exon 19) in two *RET* M918T–positive cases. One high-VAF *HRAS*-mutated patient also harbored a low-frequency *NRAS* variant. Finally, four patients showed isolated low-VAF oncogenic variants affecting *KMT2A* (two cases), *PTEN*, *PIK3CA*, or *TP53* (Fig. 2b).

### Distribution and prevalence of somatic variants in *RET*-positive, *RAS*-positive, and *RET/RAS*-negative patient groups

The distribution of oncogenic alterations identified in the 94 sMTC patients is summarized in Table S3. The majority harbored a single variant, predominantly affecting *RET* or members of the *RAS* gene family. Specifically, 47% (44/94) harbored a single oncogenic variant, 36% (34/94) had two, 6% (6/94) had three, and 2% (2/94) carried five variants. Among *RET*-positive patients (n = 50), 40% (20/50) had only the *RET* driver, 52% (26/50) had co-occurring variants, and 4% (2/50) each harbored three or four additional alterations. A total of 40 additional variants were detected, most frequently in *ATM* (20%), *KMT2A* (15%), *CDKN1A* (12.5%), and *GNAS* (10%). Second *RET* variants occurred in 10%, *PIK3CA* in 7.5%, and single-case alterations involved *GCM2, GNA11, KRAS, TP53, PTEN, TRPV5, MEN1,* and *VHL*.

Among *RAS*-positive patients (n = 27), 60% (16/27) carried a single *RAS* mutation, 26% (7/27) had one additional variant, and 14% (4/27) had two additional alterations. *ATM* (19%) and *KMT2A* (12.5%) were the most frequent additional variants, with low-frequency *RET* variants (<2% VAF) in two cases and single-case alterations in *AIP, AKT1, CDKN1C, GCM2, MET, PTEN* and *NRAS*. In the *RET/RAS*-negative group (n = 17), 53% (9/17) had oncogenic variants: 47% (8/17) with a single variant and 6% (1/17) with two. *ATM, KMT2A,* and *GNAS* were each found in 20% of cases, and single-case variants included *MAP2K1, PTEN, STK11 and TRPV6*. The remaining 47% showed no detectable alterations.

### Novel coding variants identified in the analyzed gene panel

NGS analysis identified ten oncogenic variants that were not previously reported in publicly available databases or clinical interpretation platforms, including ClinVar, Franklin (Genoox), dbSNP, and COSMIC at the moment of the study (variants are detailed in Table S4).

### Correlation between mutational status and clinical-pathological features

Patients were stratified into four groups according to *RET* and *RAS* status (*RET* M918T, other *RET* mutations, *RAS* mutations, and non-*RET*/non-*RAS*) to assess genotype–phenotype associations (Table 2). The contribution of *RET* and *RAS* to tumor development was further evaluated by correlating VAF with clinical and pathological features. *RET* mutations (M918T and others) were significantly associated with higher stages at diagnosis (III–IV vs I–II), worse clinical outcomes, disease-related death, and high-grade MTC (all p < 0.01) (Table 2). Conversely, *RAS* mutations were associated with a lower rate of disease-related death (p = 0.04) and low-grade MTC (p = 0.03).

**Table 2:** Clinicopathological Characteristics of Sporadic MTC Patients Stratified by Somatic Mutation Status.

Characteristics	RET M918T	RET other	RAS	Non-RET/non-RAS	*p* Value
**Patients**					
**Sex**					0.08
Female 59/94 (62.8%)	14/24 (58.3%)	12/26 (46.2%)	19/27 (70.4%)	14/17 (82.4%)	
Male 35/94 (37.2%)	10/24 (41.7%)	14/26 (53.8%)	8/27 (29.6%)	3/17 (17.6%)	
**Age at diagnosis (IQR)**	60.1 (22.8–65.6)	58.7 (32.7––65.6)	64.0 (52.0–68.1)	57.7 (28.1–75.1)	0.7
**Outcome**					0.04
Cured (Ct undetectable) 45/94 (47.9%)	9/24 (37.5%)	10/26 (38.5%)	15/27 (55.6%)	11/17 (64.7%)	
Not-cured (biochemical or structural disease) 40/94 (42.6%)	9/24 (37.5%)	13/26 (50.0%)	12/27 (44.4%)	6/17 (35.3%)	
Disease-related death 9/94 (9.6%)	6/24 (25.0%)	3/26 (11.5%)	0	0	
**Disease-related death**					<0.01
No 85/94 (90.4%)	18/24 (75.0%)	23/26 (88.5%)	27/27	17/17	
Yes 9/94 (9.6%)	6/24 (25.0%)	3/26 (11.5%)	0/27	0/17	
**Stage**					0.01
I-II 59/94 (62.8%)	11/24 (45.8%)	13/26 (50.0%)	20/27 (74.1%)	15/17 (88.2%)	
III-IV 35/94 (37.2%)	13/24 (52.4%)	13/26 (50.0%)	7/27 (25.9%)	2/17 (11.8%)	
**T**					0.4
I-II 71/94 (75.5%)	15/24 (62.5%)	21/26 (80.8%)	21/27 (77.8%)	14/17 (82.4%)	
III-IV 23/94 (24.5%)	9/24 (37.5%)	5/26 (19.2%)	6/27 (22.2%)	3/17 (17.6%)	
**N**					<0.01
N0 60/94 (63.8%)	11/24 (45.8%)	13/26 (50%)	20/27 (74.1%)	16/17 (94.1%)	
N1 34/94 (36.2%)	13/24 (54.2%)	13/26 (50%)	7/27 (25.9%)	1/17 (5.9%)	
**Distant metastasis (M)**					0.08
M0 85/94 (90.4%)	19/24 (79.2%)	23/26 (88.5%)	26/27 (96.3%)	17/17	
M1 9/94 (9.6%)	5/24 (20.8%)	3/26 (11.5%)	1/27 (3.7%)	0/17	
**Grading**					<0.01
Low-grade 72/92 (78.3%)	12/22 (54.5%)	20/26 (76.9%)	25/27 (96.6%)	15/17 (88.2%)	
High-grade 20/92 (21.7%)	10/22 (45.5%)	6/26 (23.1%)	2/27 (7.4%)	2/17 (11.8%)	

**Fig. 3:**
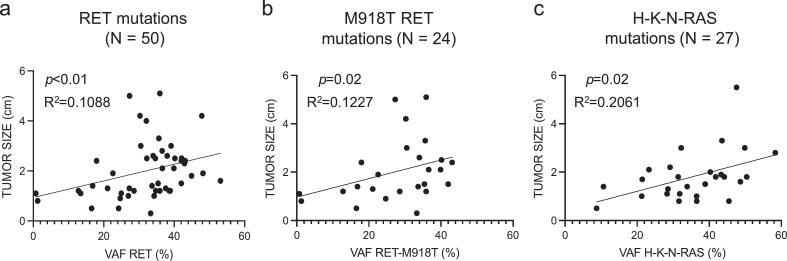
Correlation between variant allele frequency and tumor size in sMTC patients. Scatter plots showing the correlation between tumor size (cm) and the variant allele frequency (VAF) of driver mutations in sMTC. Analyses were conducted for the entire *RET*-mutated subgroup (*a*, *p* <0.01), the *RET* M918T hotspot (*b*, *p* = 0.012), and the *RAS*-mutated subgroup (including *H*-, *K*-, and *N-RAS* mutations; *c*, *p* = 0.02). All correlations were statistically significant (Spearman’s test).

In *RET*-positive cases (mean VAF 33.3%, median 34.7%, range 12.8–53.1%, IQR 27.3–39), VAF was significantly associated with tumor size (p <0.01, R^2^ = 0.1088) (Fig. 3a). This correlation remained significant in *RET* M918T tumors (mean VAF 27.88%, median 30.5%, range 20.3–36.0%, IQR 21–36; p = 0.02, R^2^ = 0.1227) (Fig. 3b). Similarly, in *RAS*-positive patients (mean VAF 35.48%, median 36.52%, range 28.5–43.5%, IQR 28.8–43.8), VAF correlated with tumor size (p = 0.02, R^2^ = 0.2061) (Fig. 3c). Higher VAF values were also observed in patients who died of disease compared with those in remission (median VAF 37.6%, IQR 25–31.4).

**Fig. 4:**
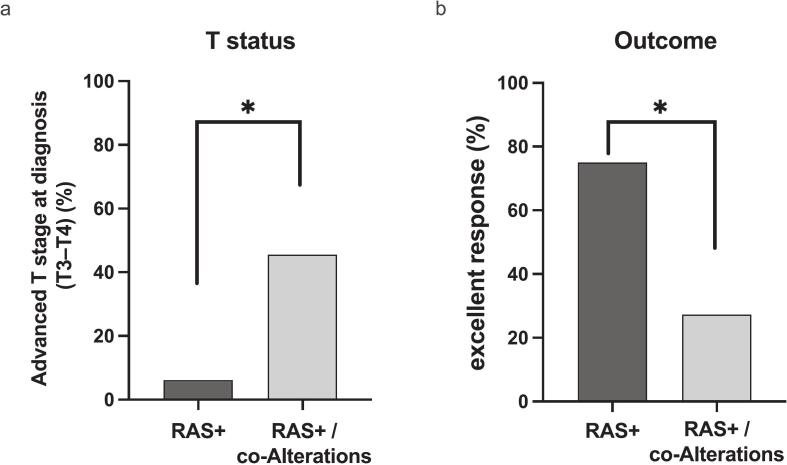
Oncogenic co-alterations identify a clinically aggressive subset of *RAS*-mutant sMTC. Patients were stratified by driver mutation status and presence of additional somatic co-alterations. (a) Advanced primary tumor stage at diagnosis (T3–T4) was significantly more frequent in *RAS*-mutant tumors with co-alterations (45.5%, 5/11) than in *RAS*-mutant tumors without co-alterations (6.2%, 1/16; χ^2^ = 5.582, *p* = 0.012). (b) An excellent biochemical response was achieved less frequently in patients with *RAS* mutations and co-alterations (27.3%, 3/11) compared with those harboring isolated *RAS* mutations (75.0%, 12/16; χ^2^ = 5.791, *p* = 0.02). No clinically meaningful differences were observed between patients with isolated *RET* mutations and those with *RET* mutations plus co-alterations across all evaluated clinical parameters. (χ square test; *: *p* < 0.05).

To further investigate the impact of additional co-occurring genomic events, patients were stratified as *RET*-only vs *RET* plus co-alterations and *RAS*-only vs *RAS* plus co-alterations. No significant clinicopathological differences were observed between *RET*-only and *RET* plus co-alteration groups (data not shown). By contrast, in the *RAS*-mutant subgroup, tumors with co-occurring alterations showed a more aggressive clinicopathological profile. Advanced T stage (T3–T4) was present in 45.5% (5/11) of cases with co-alterations versus 6.2% (1/16) with isolated *RAS* mutations (χ^2^ = 5.582, p = 0.02) (Fig. 4a). Accordingly, biochemical cure was achieved in 27.3% (3/11) of patients with co-alterations compared with 75.0% (12/16) of those harbouring RAS mutations alone (χ^2^ = 5.791, p = 0.02) (Fig. 4b). No significant differences were observed in other parameters, including tumor size, lymph node involvement, distant metastases, grade, stage, survival, or disease-free survival.

### Clinical outcomes in sMTC by driver mutation status: Kaplan–Meier and Cox regression analyses of RAS and RET mutations

*RAS* mutations were associated with longer disease-free survival (DFS), defined as undetectable Ct (log-rank p < 0.01) and with longer disease-specific survival (DSS) compared with non-*RAS* cases (log-rank p = 0.02) (Fig. S2a). Conversely, *RET* mutations correlated with shorter DFS (log-rank p < 0.0001) (Fig. S2b) and DSS (log-rank p < 0.001) (Fig. S2c).

Among molecular subgroups (*RAS*-mutated, *RET* M918T, other *RET* mutations, and non-*RET*/non-*RAS*), DSS was significantly shorter in *RET* M918T patients than in *RAS*-mutated patients (Bonferroni p = 0.01) and was also reduced in patients with other *RET* mutations compared with *RAS*-mutated cases (Bonferroni p = 0.04). A similar trend was observed for DFS, which was shorter in *RET* M918T patients (Bonferroni p = 0.02) and in those with other *RET* mutations (Bonferroni p <0.01) compared with *RAS*-mutated patients.

A multivariable survival analysis using a Cox proportional hazard regression model (stepwise), including *RET* and *RAS* somatic mutations, tumor stage, and IMTCGS grade, identified IMTCGS grade as the only independent predictor of DSS (HR 4.9, 95% CI 1.1–22.6, p = 0.04). Using the same covariates, outcome was independently influenced by *RAS* mutations (HR 0.4, 95% CI 0.2–0.8, p < 0.1) and higher stage at diagnosis (HR 3.4, 95% CI 1.8–6.2, p < 0.0001).

## Discussion

In the present study, a targeted gene panel was designed to identify somatic variants in sMTC patients using deep NGS sequencing. Genes were selected based on their involvement in neuroendocrine diseases and their critical roles in oncogenic pathways. *ATM*, *KMT2A, CDKN1A*, and *GNAS* emerged as the most frequently altered genes beyond *RET* and *RAS*, detected in 14% (13/94), 10% (10/94), 6% (6/94), and 6% (6/94) of cases, respectively. Notably, when stratifying the 94 analyzed patients into *RET*-positive, *RAS*-positive, and *RET*/*RAS*-negative groups, we observed that *ATM* and *KMT2A* were the only genes in our panel to harbor oncogenic variants across all subgroups, suggesting a secondary acquisition and a potential supportive role in MTC tumorigenesis or progression.

Loss-of-function mutations in *ATM* have previously been described in sMTC [14], [15], adrenocortical carcinoma [16], and other aggressive endocrine malignancies. In these studies, reduced *ATM* expression has been linked to metastatic potential ^15,16^. Given their critical role in DNA damage response and cell cycle regulation, the presence of *ATM* alterations, particularly among *RET*/*RAS*-negative patients, supports their putative tumor suppressor function in MTC [17], [18].

Similarly, the cyclin-dependent kinase inhibitors *CDKN1A* and *CDKN2C* play a central role in tumor suppression by modulating cell cycle checkpoints. *CDKN1A*, which encodes the p21 protein, is a key effector of p53-mediated cell cycle arrest [12]. Similarly, loss of *CDKN2C* has been linked to C-cell hyperplasia in murine models (RET2B; p18+/−) [19]. In human MTC, *CDKN2C* inactivation in combination with the *RET* M918T mutation, as described by Grubbs *et al*. [20], markedly increases tumor risk and accelerates disease progression, highlighting the role of impaired cell-cycle control in tumor dissemination [19], [20].

*KMT2A* encodes for a histone methyltransferase involved in chromatin remodeling and gene transcription[21], and its oncogenic variants were detected across all subgroups. Alterations in this gene family have been implicated in various thyroid tumors and are increasingly recognized as contributing to epigenetic dysregulation in endocrine tumorigenesis [21].

In addition, alterations in *GNAS* were identified in six cases, including both the *RET*-positive and *RET/RAS*-negative subgroups. *GNAS* encodes the Gsα subunit involved in cAMP signaling and is a well-established oncogenic driver in several endocrine tumors, particularly in the context of McCune-Albright syndrome [22] and somatotroph pituitary neuroendocrine tumors (PitNETs) [23]. While *GNAS* mutations are rare in thyroid carcinoma [23], [24], their presence in our sMTC cohort raises the possibility of them playing a broader role in tumorigenesis.

Oncogenic variants affecting key genes of the PI3K/AKT/mTOR signaling pathway were also identified across all molecular subgroups, supporting the involvement of this pathway in the dysregulation in MTC tumorigenesis and progression [25], [26], [27], [28].

Consistent with prior studies, our data confirm that *RET* and *RAS* mutations dominate the genetic landscape of sMTC, accounting for 53% (50/94) and 29% (27/94) of cases, respectively [9], [29], [30]. The *RET* M918T mutation emerged as the most frequent alteration (52% of *RET*-mutated cases), in line with its well-established role as a strong oncogenic driver associated with aggressive disease [6], [9], [31].

Although additional *RET* variants with extremely low VAFs (<5%, e.g., D631E, D567N, T1038A, and S891A) were detected in tumors already harboring established *RET* or *RAS* driver mutations, their low prevalence most likely reflects subclonal diversification rather than independent driver events [32], [33], [34], [35]. The co-occurrence of typically mutually exclusive alterations, such as *RET* and *RAS*, can be explained by distinct minor clones within the same lesion. This finding suggests that even well-characterized drivers may accumulate secondary alterations contributing to intratumoral heterogeneity [9], [35], [36].

Further analysis revealed a positive correlation between VAF percentage of driver mutations and tumor size in both *RET*- and *RAS*-mutated subgroups. These findings collectively support a model in which *RET* and *RAS* mutations act as clonal drivers of tumor expansion, with VAF reflecting the extent of clonal proliferation. Moreover, associations between *RET* mutations and shorter DFS and DSS were confirmed, whereas *RAS* mutations were associated with lower tumor grade and a significantly reduced risk of disease-related death.

Notably, extended somatic profiling revealed clinically relevant molecular heterogeneity within the *RAS*-mutant subgroup. Stratification based on the presence of additional somatic co-alterations identified significant clinicopathological differences compared with tumors harboring isolated *RAS* mutations. Specifically, RAS-mutant tumors with co-alterations were associated with a less favorable clinical profile, including higher primary tumor T status at diagnosis (T3–T4 vs T1–T2) and reduced rates of biochemical cure.

In contrast, no significant clinical differences were observed between tumors carrying isolated *RET* mutations and those harboring *RET* mutations with additional co-alterations. This observation suggests that *RET* mutations alone may be sufficient to drive an intrinsically aggressive tumor phenotype, particularly in a malignancy characterized by a relatively low mutational burden. Conversely, these findings provide evidence that *RAS*-driven tumorigenesis may require additional cooperating genomic events to acquire a more aggressive clinical behavior, as reflected by the poorer outcomes observed in *RAS*-mutant tumors with co-alterations.

Strengths of the study include its contribution to understanding the molecular landscape of MTC, particularly RAS-mutated MTC, whose underlying pathways remain poorly characterized in the literature. Additionally, we have linked these novel findings to the clinical outcome of the disease. However, we must also acknowledge the limitations of this study, primarily related to the analysis of a relatively limited number of molecular events (31) and the focus on mutations, deletions, and amplifications, without investigating other potentially relevant alterations such as gene fusions and epigenetic events.

In conclusion, targeted somatic profiling identified oncogenic alterations affecting pathways related to DNA damage repair, epigenetic regulation, cell-cycle control, and intracellular signaling, including *ATM, KMT2A, GNAS,* and *CDKN1A*.

Importantly, extended panel-based profiling uncovered clinically meaningful heterogeneity within *RAS-*mutant MTCs. Specifically, a subset of *RAS-*driven tumors harboring additional co-occurring alterations exhibited a significantly less favorable clinicopathological profile, highlighting the added value of broader genomic characterization in refining risk stratification and supporting more precise, molecular-driven clinical management.

## Author contributions

ER, AB, SB, LB, FC, GA, and GC performed the experiments.

ER, SC, SB, LB, AG, and CG analyzed the data and wrote the manuscript.

SC, IP, CC, FG, GP and MI provided patient samples and retrieved patient clinical data.

ER, SC, SB, VM, MV, and CM designed the research study.

SC, SB, GC, AG and CM critically reviewed and edited the manuscript.

CM and MV acquired funds for the research.

## CRediT authorship contribution statement

**Edoardo Ruggeri:** Writing – original draft, Software, Methodology, Conceptualization. **Simona Censi:** Visualization, Supervision. **Loris Bertazza:** Visualization, Methodology, Investigation. **Andrea Benetti:** Methodology. **Fausto Cortese:** Data curation. **Ilaria Piva:** Resources. **Cristina Clausi:** Resources. **Vincenzo Marotta:** Validation. **Francesca Galuppini:** Resources. **Alessandra Giannella:** Formal analysis, Data curation. **Gianmaria Pennelli:** Resources. **Maurizio Iacobone:** Resources. **Mario Vitale:** Validation, Resources. **Giulio Ceolotto:** Software, Data curation, Conceptualization. **Susi Barollo:** Supervision, Methodology, Investigation, Data curation. **Caterina Mian:** Visualization, Supervision, Project administration, Conceptualization.

## Funding

This work was supported by the PRIN 2022 Project (code: 20225WNCYS), which also funded a fellowship awarded to E.R. The funders had no role in the study design, the data collection and analysis, the decision to publish or the preparation of the manuscript.

## Declaration of competing interest

The authors declare that they have no known competing financial interests or personal relationships that could have appeared to influence the work reported in this paper.

## Appendix A Supplementary data

Supplementary Data 1 Somatic alterations according to RET/RAS status.

Supplementary Data 2 Clinical outcomes according to driver mutation status in sMTC. Kaplan–Meier analysis of disease-specific survival (DSS) and disease-free survival (DFS) in 94 sMTC patients, stratified by mutation status. (a) RAS-mutant patients exhibited longer DSS (log-rank test, p = 0.02) than RAS wild-type patients. (b) RET mutated patients exhibited a shorter DFS (p < 0.0001) and (c) shorter DSS (p < 0.001) than RET wild-type patients.
