# Supplementary figures and images for: Somatic genomic profiling reveals clinically relevant heterogeneity in RAS-mutant sporadic medullary thyroid carcinoma
Source: J Clin Transl Endocrinol. 2026 Apr 28;44:100442. doi: 10.1016/j.jcte.2026.100442 (PMC13158359; doi:10.1016/j.jcte.2026.100442)

**a**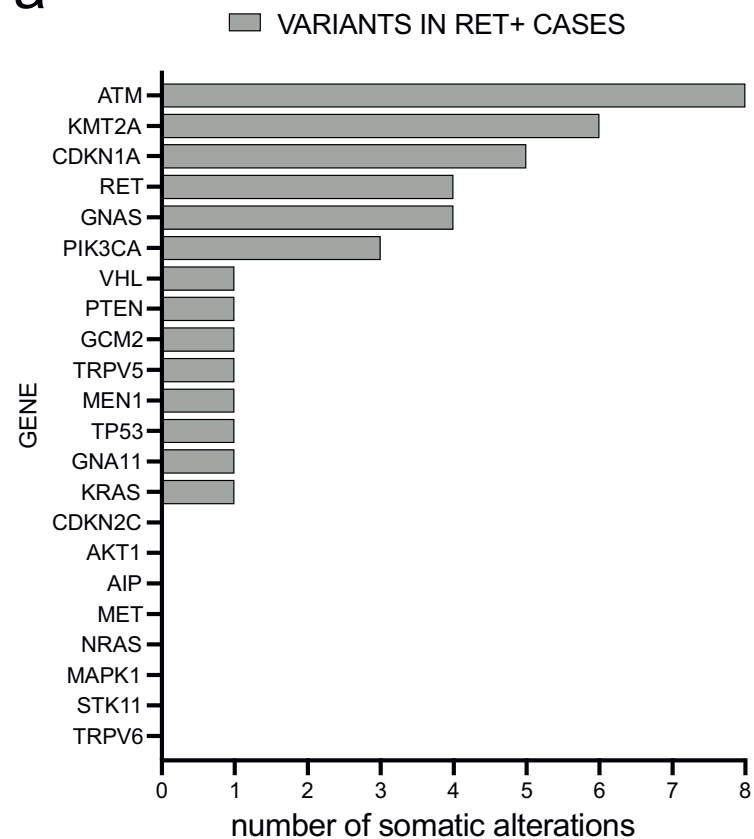**b**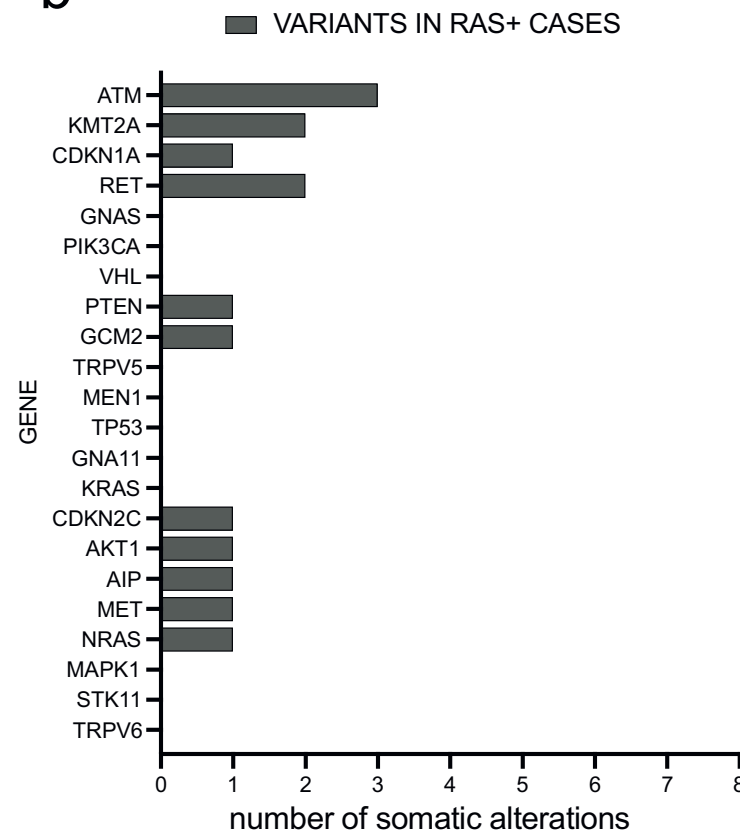**c**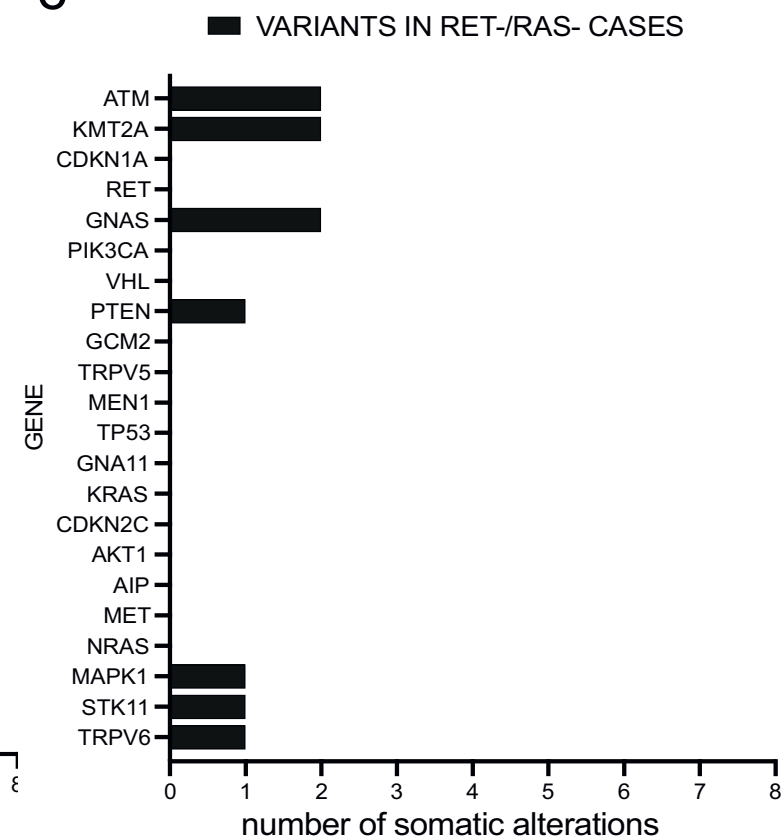

Supplement: Supplementary Data 1 — Somatic alterations according to RET/RAS status. [file mmc1.pdf]
