# Supplementary material for: Somatic genomic profiling reveals clinically relevant heterogeneity in RAS-mutant sporadic medullary thyroid carcinoma
Source: J Clin Transl Endocrinol. 2026 Apr 28;44:100442. doi: 10.1016/j.jcte.2026.100442 (PMC13158359; doi:10.1016/j.jcte.2026.100442)
Supplement: Supplementary Data 2 — Clinical outcomes according to driver mutation status in sMTC. Kaplan–Meier analysis of disease-specific survival (DSS) and disease-free survival (DFS) in 94 sMTC patients, stratified by mutation status. (a) RAS-mutant patients exhibited longer DSS (log-rank test, p = 0.02) than RAS wild-type patients. (b) RET mutated patients exhibited a shorter DFS (p < 0.0001) and (c) shorter DSS (p < 0.001) than RET wild-type patients. [file mmc2.pdf]

Figure S2

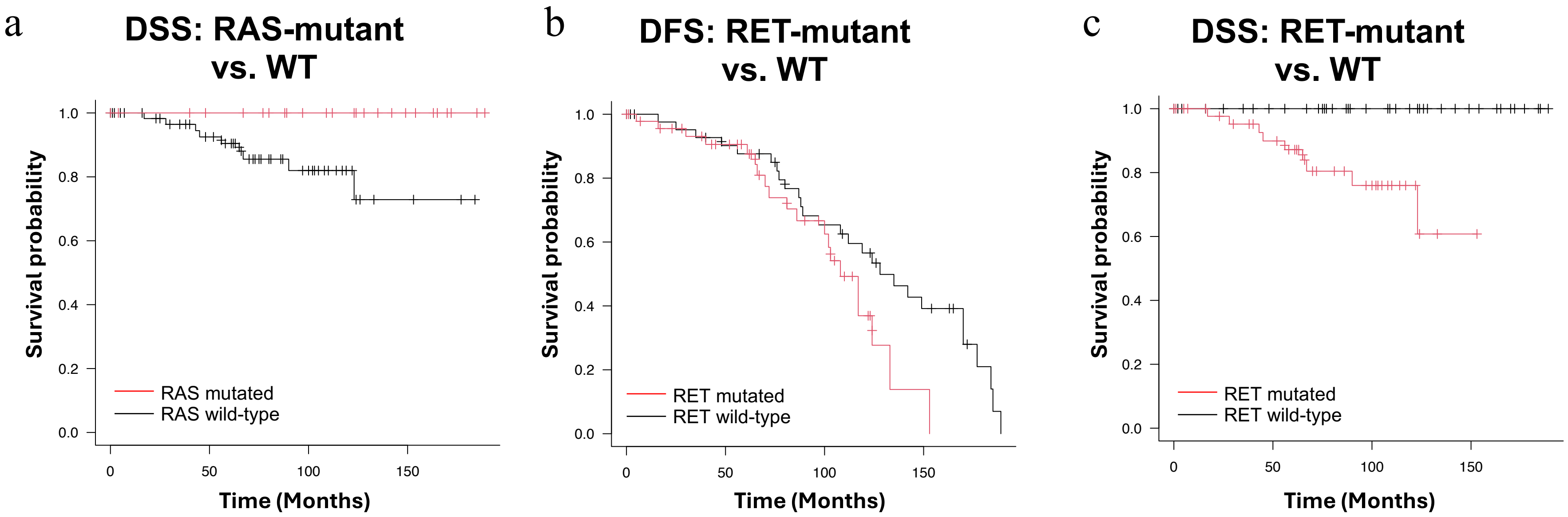

Number at risk

Wild-type	65	46	22	3
Mutated	27	23	17	9

Number at risk

Wild-type	44	35	23	11
Mutated	48	34	16	1

Number at risk

Wild-type	44	35	23	11
Mutated	48	34	16	1
