# Supplementary material for: Somatic genomic profiling reveals clinically relevant heterogeneity in RAS-mutant sporadic medullary thyroid carcinoma
Source: J Clin Transl Endocrinol. 2026 Apr 28;44:100442. doi: 10.1016/j.jcte.2026.100442 (PMC13158359; doi:10.1016/j.jcte.2026.100442)
Supplement: Supplementary Data 3 [file mmc3.docx]

Table S1. Probe-based detection assay ID.

| **Gene Name** | **Gene Symbol** | **TaqMan probe: Assay ID** |
| --- | --- | --- |
| calcitonin related polypeptide alpha | *CALCA* | Hs01100741_m1 |
| thyroglobulin | *TG* | Hs00174974_m1 |
| actin beta | *ACTB* | Hs99999903_m1 |
