# Supplementary material for: Somatic genomic profiling reveals clinically relevant heterogeneity in RAS-mutant sporadic medullary thyroid carcinoma
Source: J Clin Transl Endocrinol. 2026 Apr 28;44:100442. doi: 10.1016/j.jcte.2026.100442 (PMC13158359; doi:10.1016/j.jcte.2026.100442)
Supplement: Supplementary Data 4 [file mmc4.docx]

| **Gene Symbol (HGNC approved)** | **NM Code** |
| --- | --- |
| ***AIP*** | NM_003977.2 |
| ***AP2S1*** | NM_004069.6 |
| ***CASR*** | NM_000388.4 |
| ***CDC73*** | NM_024529.4 |
| ***CDKN1A*** | NM_078467.2 |
| ***CDKN1B*** | NM_004064.4 |
| ***CDKN2B*** | NM_004936.3 |
| ***CDKN2C*** | NM_001262.3 |
| ***CDKN2D*** | NM_001800.4 |
| ***GCM2*** | NM_004752.4 |
| ***GNA11*** | NM_002067.5 |
| ***GNAS*** | NM_000516.7 |
| ***MEN1*** | NM_130799.2 |
| ***TRPV6*** | NM_018646.6 |
| ***TRPV5*** | NM_019841.7 |
| ***RET*** | NM_020975.6 |
| ***KRAS*** | NM_033360.4 |
| ***HRAS*** | NM_005343.4 |
| ***NRAS*** | NM_002524.5 |
| ***TP53*** | NM_000546.5 |
| ***MET*** | NM_000245.4 |
| ***STK11*** | NM_000455.5 |
| ***ARID2*** | NM_152641.4 |
| ***SETD2*** | NM_014159.7 |
| ***KMT2A*** | NM_001197104.2 |
| ***KMT2C*** | NM_170606.3 |
| ***PIK3CA*** | NM_006218.4 |
| ***AKT1*** | NM _001382430.1 |
| ***CTNNB1*** | NM_001904.4 |
| ***PTEN*** | NM_000314.8 |
| ***ATM*** | NM_000051.4 |
| ***EIF1AX*** | NM_001412.4 |
| ***MAP2K1*** | NM_002755.4 |
| ***VHL*** | NM_000551.4 |

Table S2.

Targeted gene panel.
