# Supplementary material for: Somatic genomic profiling reveals clinically relevant heterogeneity in RAS-mutant sporadic medullary thyroid carcinoma
Source: J Clin Transl Endocrinol. 2026 Apr 28;44:100442. doi: 10.1016/j.jcte.2026.100442 (PMC13158359; doi:10.1016/j.jcte.2026.100442)
Supplement: Supplementary Data 5 [file mmc5.docx]

**Table S3.** Distribution of somatic variants detected in the sMTC population. Numbers in square brackets indicate VAF expressed as a percentage.

| **sample ID** | **1 ALTERATION** | **sample ID** | **2 ALTERATIONS** | **sample ID** | **3 ALTERATIONS** | **sample ID** | **5 ALTERATIONS** |
| --- | --- | --- | --- | --- | --- | --- | --- |
| **1** | GNAS P459R [36,55] | **2** | RET A883F [38,03];  ATM D1853N [13,60] | **15** | KRAS G12R [40,30];  CDKN1A S130F [8,95];  KMT2A A67del [1,74] | **54** | RET M918T [27,33];  RET D567N [48,0];  PTEN A137S [48,88];  ATM D1853N [50,22];  GNAS E114_T115insSerE [46,65] |
| **6** | RET A883F [24,97] | **4** | RET D898_E901del [39,12]; ATM L1420F [44,55] | **29** | HRAS M72I [45,47];  RET M918T [1,24];  AKT1 M118L [51,44] | **90** | RET C634R [42,12];  PIK3CA I391M [26,65];  CDKN1A S31R [48,733];  KMT2A W1474fs [10,64];  TP53 R213G [1,20] |
| **7** | HRAS K117N [33,88] | **9** | RET D631_L633delinsE. [32,2];  CDKN1A C117Y [48,93] | **46** | HRAS Q61K [21,29];  NRAS Q61R [0,55];  ATM V3020L [19,55] |  |  |
| **8** | HRAS Q61K [48,68] | **17** | HRAS Q61L [47,60];  CDKN2C L89fs [8,088] | **89** | RET C634S [34,66];  CDKN1A S31R [36,82];  GNAS T415_G423del [35,74] |  |  |
| **10** | HRAS Q61R [28,54] | **22** | RET C634W [34,27];  PIK3CA E545K [1,70] | **103** | RET M918T [22,58];  PIK3CA I391M [50,74];  GNAS P459R [59,54] |  |  |
| **11** | RET M918T [30,50] | **23** | RET C634Y [36,57];  KMT2A P245fs [30,37] | **69** | HRAS Q61K [28,34];  RET M918T [0,73];  KMT2A P2155S [46,37] |  |  |
| **12** | RET M918T [12,82] | **25** | HRAS Q61K [50,41];  MET T273N [43,88] |  |  |  |  |
| **13** | RET M918T [35,66] | **27** | RET M918T [30,27];  KMT2A P245fs [8,82] |  |  |  |  |
| **14** | HRAS Q61R [36,52] | **30** | HRAS G12R [23,28];  ATM P604S [46,53] |  |  |  |  |
| **19** | HRAS Q61R [41,83] | **39** | RET A883F [32,09];  KMT2A A67del [1,72] |  |  |  |  |
| **20** | KRAS G12R [32,22] | **42** | HRAS Q61K [21,42];  ATM V3020L [21,75] |  |  |  |  |
| **24** | RET M918T [43,12] | **43** | RET M918T[35,43];  MEN1 D418N [6,03]; |  |  |  |  |
| **28** | NRAS Q61R [31,39] | **44** | RET M918T [42,03];  KRAS D108Y [55,96]; |  |  |  |  |
| **36** | KRAS G12R [43,54] | **45** | RET C634Y [37,58];  RET S891A [2,98] |  |  |  |  |
| **37** | HRAS Q61R [36,56] | **50** | RET L629_L633del [30,0]; ATM G2023R [48,86] |  |  |  |  |
| **38** | HRAS G60D [49,89] | **51** | KRAS Q61R [8,75];  AIP R304Q [49,46] |  |  |  |  |
| **40** | RET M918T [21,07] | **53** | MAP2K1 G236E [47,99]; KMT2A A67del [1,37] |  |  |  |  |
| **47** | RET M918T [37,51] | **56** | RET M918T [39,92];  ATM D1853N [48,17] |  |  |  |  |
| **48** | STK11 S404F [51,48] | **58** | RET M918T [35,92];  VHL C77fs [19,82] |  |  |  |  |
| **52** | RET M918T [40,1]; | **61** | KRAS Q61H [58,33];  PTEN A252T [45,60] |  |  |  |  |
| **55** | NRAS Q61K [10,66] | **64** | RET A883F [34,74];  ATM Y583S [51,35] |  |  |  |  |
| **57** | RET M918T [33,68] | **70** | ATM E2936Q [49,55];  RET M918T [16,856] |  |  |  |  |
| **59** | RET A883F [38,83] | **71** | HRAS Q61R [43,32];  GCM2 D107N [50,82] |  |  |  |  |
| **60** | RET M918T [24,67] | **76** | RET C618R [44,91];  GCM2 N503S [48,89] |  |  |  |  |
| **62** | ATM R2719H [49,69] | **81** | RET D631E [14,24];  RET P496_D499del [13,43] |  |  |  |  |
| **63** | KRAS G12R [29,14] | **86** | RET M918T [36,62];  GNA11 R92W [48,62] |  |  |  |  |
| **65** | RET I590_G607de [26,98] | **87** | RET M918T [33,32];  RET T906A [52,29] |  |  |  |  |
| **66** | HRAS G13R [31,82] | **88** | RET M918T [28,57];  CDKN1A C117Y [48,45] |  |  |  |  |
| **67** | RET M918T [34,02] | **92** | RET C634Y [42,90];  CDKN1A S31R [48,96] |  |  |  |  |
| **73** | RET M918T [16,56] | **93** | RET C634R [47,83];  GNAS R600G [48,33] |  |  |  |  |
| **74** | PTEN L1P [0,85] | **94** | RET M918T [35,931];  ATM K1964E [46,479] |  |  |  |  |
| **78** | ATM K1435T [45,73] | **95** | RET A883F [24,21];  TRPV5 A102T [48,50] |  |  |  |  |
| **79** | GNAS P459R [35,51] | **101** | RET D898_E901del [27,251];  KMT2A P2774A [49,33] |  |  |  |  |
| **80** | TRPV6 A18S [49,06] | **72** | RET C630R [42,02];  KMT2A G3585V [4,51] |  |  |  |  |
| **82** | RET C634W [38,60] |  |  |  |  |  |  |
| **84** | RET C634Y [53,119] |  |  |  |  |  |  |
| **85** | KRAS G12V [38,89] |  |  |  |  |  |  |
| **91** | RET C618G [48,15] |  |  |  |  |  |  |
| **96** | KRAS G12R [31,62] |  |  |  |  |  |  |
| **106** | HRAS Q61K [44,18] |  |  |  |  |  |  |
| **18** | RET M918T [17,94] |  |  |  |  |  |  |
| **21** | RET M918T [31,45] |  |  |  |  |  |  |
| **31** | RET M918T [20,88] |  |  |  |  |  |  |
| **108** | KMT2A A11V [5,99] |  |  |  |  |  |  |
