# Supplementary material for: Somatic genomic profiling reveals clinically relevant heterogeneity in RAS-mutant sporadic medullary thyroid carcinoma
Source: J Clin Transl Endocrinol. 2026 Apr 28;44:100442. doi: 10.1016/j.jcte.2026.100442 (PMC13158359; doi:10.1016/j.jcte.2026.100442)
Supplement: Supplementary Data 6 [file mmc6.docx]

**Table S4**. Novel somatic alterations identified by NGS analysis.

| **ID**  **No.** | **ZYGOSITY** | **Homo sapiens (GRCh38.108) _Gene** | **VAF %** | **Amino acid change** | **Coding region change** | **COSMIC** |
| --- | --- | --- | --- | --- | --- | --- |
| 23 | Heterozygous | *KMT2A* | 30,37 | P245fs | c.732dupA | NEW |
| 27 | Heterozygous | *KMT2A* | 8,82 | P245fs | c.732dupA | NEW |
| 72 | Heterozygous | *KMT2A* | 4,51 | G3585V | c.10754G>T | NEW |
| 108 | Heterozygous | *KMT2A* | 5,99 | A11V | c.32C>T | NEW |
| 42 | Heterozygous | *ATM* | 21,75 | V3020L | c.9058G>T | NEW |
| 46 | Heterozygous | *ATM* | 19,54 | V3020L | c.9058G>T | NEW |
| 81 | Heterozygous | *RET* | 13,43 | P496_D499del | c.1485_1496delTCCACTGTGCGA | NEW |
| 17 | Heterozygous | *CDKN2C* | 8,08 | L89fs | c.265_266delTT | NEW |
| 54 | Heterozygous | *GNAS* | 46,65 | E114_T115insSerE | c.-400_-395dupGCGAGA | NEW |
| 29 | Heterozygous | *AKT1* | 51,44 | M118L | c.352A>T | NEW |
